# Beyond the Bench: Environmental Health Nursing: Putting Knowledge into Practice

**DOI:** 10.1289/ehp.114-a283

**Published:** 2006-05

**Authors:** Tanya Tillett

Training that delivers a complete picture of environmental dangers faced by communities is critical in helping environmental health nurses cultivate skills that go beyond basic health care. Now the Community Outreach and Education Cores (COECs) at the University of Wisconsin (UW)–Milwaukee Marine and Freshwater Biomedical Sciences Center and the Harvard NIEHS Center for Environmental Health have joined forces to take environmental health nurse training to the next level by combining didactic and onsite practice teaching methods into one integrated learning experience.

This learning experience includes a site visit within the larger context of a two-day conference. “These intense conferences are intended to provide some in-depth education for public health nurses who are on the front line of environmental health in communities everywhere, and for nursing faculty, who need to understand environmental health in order to incorporate that content into nursing education at all levels,” says Jeanne Hewitt, director of the UW–Milwaukee COEC. Attendees receive continuing education units, which are authorized by the American Nursing Association and count toward the professional education that is required of nurses in some states.

The first conference was held in July 2005 at the Harvard School of Public Health (HSPH), and focused on helping academic and practicing nurses bring environmental health concepts into the classroom, practice, and policy arenas. With help from HSPH visiting scholar Stephanie Chalupka of the University of Massachusetts Lowell, the COECs designed a conference program that reflected the complexity and interrelationship of environmental health issues as well as the scope and nature of the practice of public health nursing. Activities included lectures, open discussions, hands-on computer work, and project development work group sessions focusing on the toxicology of organochlorines, the epidemiology of trichloroethylene, the existence of disease clusters, and the usefulness of geographic information system mapping technology in community health research and risk assessment.

The 2005 conference also included a teaching experiment that served as a bridge between the instructional segments and a site visit to the Wells G & H Superfund site in Woburn, Massachusetts (these two municipal wells were found to be contaminated with industrial waste in 1979). To illustrate the fate and transport of toxicants through different soils, the COECs used experiments created by staff from the Edgerton Educational Center at the Massachusetts Institute of Technology. One activity used simulated lake water, a surrogate “toxicant” (colored candy), and four containers, each layered with different amounts of clay and medium, coarse, and fine sand. The experiment revealed flow rates in various soils and showed how clay forms a barrier to flow. Once the nurses had an understanding of the properties of different soils, they visited the Superfund site to examine the contaminated soil there. The interactive design of the conference allowed them to apply the latest environmental health information directly to community analysis.

These conferences help nurses develop skills that respond to current environmental challenges that threaten the public’s health. As Ann Backus, director of the Harvard COEC, points out, “Today’s health problems stem not only from communicable diseases and other concerns such as nutrition, maternal and child health, disasters, and war-related injuries, but also from contamination of our water, soil, and air—the ‘commons’ we count on to keep us healthy rather than make us ill. We need now to usher in a new era of public health nursing which will be known for its application of the concepts and competencies in environmental and public health nursing to the prevention of illness in the population through stewardship of the environment. We need also to re-energize the demand for public health nurses who are competent in environmental health.”

A second conference, scheduled for 1–4 August 2006 at UW–Milwaukee, will focus on the human health effects of mercury in the environment. For more information and online registration, see http://www.uwm.edu/Dept/MFB/nursingconference/.

## Figures and Tables

**Figure:**
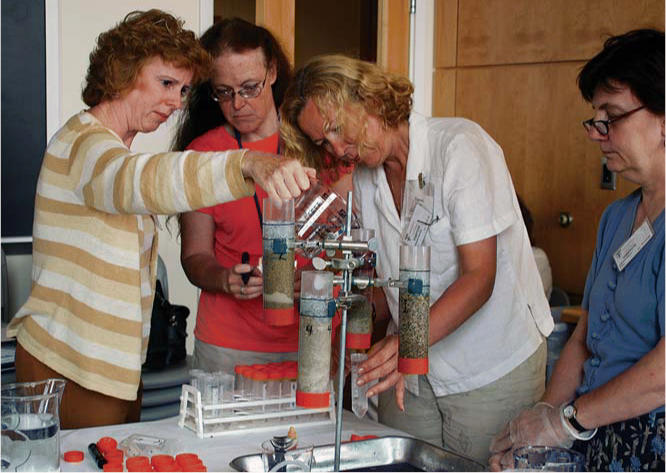
Field prep. Public health nurses study how contaminants travel through different types of soil in preparation for a visit to a Superfund site as part of a two-day conference.

**Figure:**
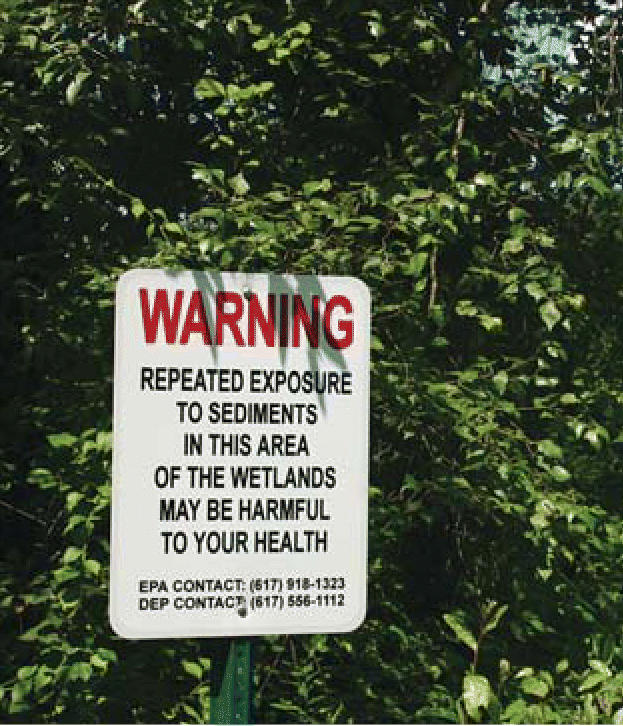
The real deal. Health warnings mark the Wells G & H Superfund site visited by the conference participants.

